# Post Partum Hemorrhage*Read at Palestine meeting East Texas Medico-Chirurgical Society, July 6, 1900.

**Published:** 1900-09

**Authors:** S. Merriwether

**Affiliations:** Grapeland, Texas


For the Texas Medical Journal.

### Post Partum Hemorrhage.*

S. MERRIWETHER, M. D., GRAPELAND, TEXAS.

This accident is one of the most alarming conditions that may arise in the progress of labor. As the name implies, it occurs during or after the third stage of labor. The occurrence of its symptoms is often sudden, falling upon the accoucheur and attendants like a thunderbolt, producing frequently utter dismay and utter demoralization; completely disarming the accoucheur, thereby ren-

*Read at Palestine meeting East Texas Medico-Chirurgical Society, July 6, 1900.

dering him unable to give that immediate and prompt aid, so essential in this condition to ward off the impending crisis.

Post partum hemorrhage may be uterine, vaginal or vulvar. The first variety is that of which this paper will treat; the others not being so frequent, and treated upon entirely different principles. The uterine variety is most frequent, and yet its causes are entirely preventable; and when it does occur can be accepted as due to ignorance or neglect in most cases. It is remarkable how few cases we have. Hundreds of cases of labor in this country have been attended by midwives, without this accident occurring, who knew absolutely nothing in regard to its prevention. This demonstrates the power of the uterus in a majority of cases to present firm normal contractions. The cause of post partum hemorrhage is uterine inertia, and is situated at the placental site, when uterine. The symptoms are usually very striking and well known to all. The sighing, death-like pallor, dilated pupils, restlessness, tossing of arms, dimness of vision, all present a picture that once seen can never be forgotten or mistaken. This is a time in which the obstetrician should be cool, calm and collected. Quick, prompt action, based upon wise decision, is essential to the salvation of his patient. The first thing to do, when serious hemorrhage is upon us, is to administer a stimulant. Sulphuric ether, strychnine, and then ergot, which should be given hypodermically. No obstetrician should be without his hypodermic syringe and tablets of strychnine, ergotin and glonoin. Right here I wish to say, that we have no more potent, prompt and reliable stimulant than strychnine. Potter says that strychnine exalts all the functions of the spinal cord—reflex-motor, vaso-motor and sensory. Prof. Jno. M. Shaller, of Cincinnati, says: "Strychnine is a remedy which, in its effects upon the nervous system, closely resembles the action of electricity. There is better receptivity and conductivity. There is increase of nervous energy, and this energy is imparted to the voluntary and involuntary muscles, causing them to respond and contract with more vigor." Strychnine in uterine inertia is one of those friends in need, that is never found wanting. It always "stands by us," and never fails. So then in this emergency, give 1-30 grain strychnine nitrate, or 1-20 grain, or even 1-15 if the urgency of the symptoms should demand it. Then ergot, as soon as possible. Parke, Davis & Co.'s aseptic ergot, or tablets of ergotine, prepared by the Abbott Alkaloidal Co., Chicago, are reliable and can be used subcutaneously, without fear of abscess. Then, if the hemorrhage is serious and death impending, we should not hesitate to introduce the hand, rendered aseptic. I use my right hand, placing the left over the fundus, and vigorously kneading it, at the same time emptying the cavity of its contents. This usually produces firm contractions, thereby checking the hemorrhage.

Prof. Miller, author of *"Miller's Obstetrics,"* in 1858 recognized this procedure as the only safe process in serious hemorrhage following labor. He first suggests, if the hemorrhage is slight, kneading of the uterus through the abdominal wall, cold applications, etc.; but if the flow continues and becomes serious, the hand and nothing but the hand, should be relied upon to bring on the necessary contractions. He also says: "You can give ergot, provided you put no trust in it." This, however, was before the days of hypodermic medication.

There are other remedies of which I have not spoken. Such as the application of styptics to the internal surface of womb, ice water, or ice itself. Hot water, vinegar, uterine and aortic compression, and last, the tampon,—which should always be introduced into the cavity itself. All of these are useful and can be tried.

This brings me to the question of prevention of hemorrhage, which should be divided into means adopted before labor begins; in the last months of gestation, and those after the commencement of labor and its completion.

We know that the period of gestation is too often neglected by the physician from a sense of modesty on the part of the patient or neglect by the physician. The history of every case that we are engaged to attend should be obtained, and its condition carefully studied and proper means adopted to carry the patient safely through this trying period. Of course, hundreds of cases need no treatment, but many do; not only for the prevention of hemorrhage but for other conditions. Shaller says: "Post partum hemorrhage should be anticipated when in previous labors uterine contractions have been feeble and loss of blood considerable. In these cases strychnine should be administered more or less continuously during the entire period of gestation. Two or three granules of the arsenite 1-134 gr. may be given three times a day for a month; then small doses of iron and quinine for a while, followed again by the hypophosphite strychnia for several weeks."

When there is enfeebled conditions of the muscular and nervous systems with loss of muscular power, I am of opinion that this procedure would give good results.

During labor, the plan that I have always followed has given good results, else I have been exceedingly fortunate in my cases. If at any time during the process of labor there is uterine inertia to contend with, I give strychnine, and as the second stage is being completed, in all cases I place the hand upon the fundus, following it down with firm pressure, thus insuring good contractions and also aiding in the expulsion of the placenta. With this procedure I very rarely have to introduce my hand to remove the placenta, and very seldom have had hemorrhage.

